# Surface Heat Balance Analysis of Tainan City on March 6, 2001 Using ASTER and Formosat-2 Data

**DOI:** 10.3390/s8096026

**Published:** 2008-09-26

**Authors:** Soushi Kato, Yasushi Yamaguchi, Cheng-Chien Liu, Chen-Yi Sun

**Affiliations:** 1 Earth Dynamic System Research Center, National Cheng Kung University, No. 1, Ta-Hsueh Road, Tainan 701, Taiwan. E-mail: ccliu88@mail.ncku.edu.tw; 2 Department of Earth and Environmental Sciences, Graduate School of Environmental Studies, Nagoya University, Nagoya, Japan. E-mail: yasushi@nagoya-u.jp; 3 Institute of Satellite Informatics and Earth Environment, Department of Earth Sciences, National Cheng Kung University, Tainan, Taiwan; 4 Department of Landscape Architecture, National Chin-Yi University of Technology, Taichung, Taiwan. E-mail: sunje.tw@yahoo.com.tw

**Keywords:** ASTER, Formosat-2, land surface classification, storage heat flux

## Abstract

The urban heat island phenomenon occurs as a mixed result of anthropogenic heat discharge, decreased vegetation, and increased artificial impervious surfaces. To clarify the contribution of each factor to the urban heat island, it is necessary to evaluate the surface heat balance. Satellite remote sensing data of Tainan City, Taiwan, obtained from Terra ASTER and Formosat-2 were used to estimate surface heat balance in this study. ASTER data is suitable for analyzing heat balance because of the wide spectral range. We used Formosat-2 multispectral data to classify the land surface, which was used to interpolate some surface parameters for estimating heat fluxes. Because of the high spatial resolution of the Formosat-2 image, more roads, open spaces and small vegetation areas could be distinguished from buildings in urban areas; however, misclassifications of land cover in such areas using ASTER data would overestimate the sensible heat flux. On the other hand, the small vegetated areas detected from the Formosat-2 image slightly increased the estimation of latent heat flux. As a result, the storage heat flux derived from Formosat-2 is higher than that derived from ASTER data in most areas. From these results, we can conclude that the higher resolution land coverage map increases accuracy of the heat balance analysis. Storage heat flux occupies about 60 to 80% of the net radiation in most of the artificial surface areas in spite of their usages. Because of the homogeneity of the building roof materials, there is no contrast between the storage heat flux in business and residential areas. In sparsely vegetated urban areas, more heat is stored and latent heat is smaller than that in the forested suburbs. This result implies that density of vegetation has a significant influence in decreasing temperatures.

## 1. Introduction

The urban heat island effect is the temperature increase according to urbanization and has been studied for about four decades. The urban heat island phenomenon occurs as a mixed result of anthropogenic heat discharge, decreased vegetation cover, and increased use of artificial impervious surface materials. These factors modify the heat balance at the land surface and eventually raise the atmospheric temperature. To clarify the contribution of each factor to the urban heat island, it is necessary to evaluate the surface heat balance. Kato and Yamaguchi [1] estimated surface heat fluxes in an urban area of Nagoya, Japan, by using visible to thermal infrared data observed by Terra ASTER and Landsat ETM+. Modifying the proposed method, The same authors [2] introduced storage heat flux, *ΔG*, in order to evaluate the heat storage and discharge of urban surfaces. Their results of estimated heat fluxes are reasonable compared with previous ground measurement data in other cities [3-8]. However, because the highest spatial resolution of ASTER sensors is 15 m for visible and near-infrared radiometer (VNIR), there is a limitation in detecting small objects in urban areas. The limitation of the spatial resolution is one of the major error sources of heat flux estimation because Kato and Yamaguchi [1, 2] assigned some important parameters according to surface types classified from satellite images. In order to reduce such error induced by mislabeled surface types, the authors used Formosat-2 data in combination with ASTER data in this study. Because of the 8m spatial resolution of Formosat-2 multispectral data, we can identify surface types with a pixel area of 3.5 times higher resolution than that of ASTER VNIR data. Moreover, spectral coverage of the blue band of Formosat-2 data is another advantage in classifying the land surface. These advantages could eliminate misclassifications caused by mixed pixels and similarities of spectral patterns. Since surface heat fluxes are estimated in the 90m spatial resolution of ASTER thermal-infrared radiometer (TIR), we can evaluate intra-pixel heterogeneity of surface coverage more clearly by Formosat-2 than ASTER data. This paper presents a case study of surface heat balance that was estimated based on surface classification maps from ASTER and Formosat-2 data in Tainan City, Taiwan on March 6, 2001. Then, the heat fluxes are compared to evaluate the sensitivity of spatial resolution and accuracy of surface classification maps against our procedure of heat balance estimation. Moreover, we interpret the characteristics of surface heat balance in Tainan City based on heat fluxes estimated from ASTER with Formosat-2 data.

## 2. Theory and Estimation Methods of Surface Heat Balance in Urban Areas

In this study, based on the same assumption used by Kato and Yamaguchi [2], who estimated the surface heat balance in an urban area by using ASTER and Formosat-2 data. For an urban surface, the absorbed net radiation and the anthropogenic heat discharge should balance the outgoing fluxes of sensible heat, latent heat and ground heat when advection is negligible,

(1) $$R_{n}+A=H+LE+G$$

where *R_n_* is the net radiation, *A* is the anthropogenic heat discharge, *H* is the sensible heat flux, *LE* is the latent heat flux, and *G* is the ground heat flux. The net radiation is the sum of the absorbed shortwave and longwave radiation. The absorbed shortwave radiation is the difference between incident shortwave radiation from the sun and reflected shortwave radiation. The net absorbed longwave radiation is determined from atmospheric emissions absorbed by the surface and emitted longwave radiation. The sensible heat and latent heat fluxes are the energy transported into the atmosphere by turbulent flow. Sensible heat increases the atmospheric temperature, while latent heat is produced by transpiration of vegetation and evaporation of land surface water, contributing to limiting surface and atmospheric temperature increases under a given net radiation. During the day, ground heat is conducted into the ground, because the surface temperature is generally higher than the underground temperature. Heat stored in the ground during the daytime is conducted to the atmosphere at night. The anthropogenic heat discharge increases the heat budget as well as the net radiation. Energy consumption due to human activities generates anthropogenic heat discharge in the form of sensible heat, latent heat, and ground heat. There is no anthropogenic heat discharge from natural land surfaces.

Because *G* and *A* depend on surface and subsurface materials and human activities, it is difficult to calculate *G* and *A* separately from satellite data. Therefore, Kato and Yamaguchi [2] applied *ΔG* estimated by merging *G* and *A* based on the heat balance equation (Equation (1)), which is often used in tower measurements in urban areas [3, 8], as follows:

(2) $$\Delta G=G-A=R_{n}-H-LE$$

For the case in which the storage heat flux exceeds 0 W m^-2^, i.e., when a downward heat flux exists, it can be interpreted as net heat storage in the urban canopy. In contrast, when the storage heat flux is negative, there is a net loss of heat from storage and/or anthropogenic heat discharge from urban canopy.

Theories for calculating the net radiation, sensible heat and latent heat fluxes have already been established. These three heat fluxes can be estimated by combining remote sensing and ground meteorological data, because these are the heat exchange between land surface and atmosphere. In the present study, general procedures for the calculation of these heat budgets are applied. Because estimation methods of these heat fluxes are described in Kato and Yamaguchi [2], the authors have omitted the explanation other than *H* and *LE* that are critical to the present study.

Sensible heat flux is given by

(3) $$H=\rho C_{p}\frac{T_{0}-T_{a}}{r_{a}}$$

where *ρ* is the air density in kg m^-3^, *C_p_* is the specific heat of air at constant pressure in J kg^-1^ K^-1^, *T_0_* is the surface aerodynamic temperature in K, *T_a_* is the atmospheric temperature in K, and *r_a_* is the aerodynamic resistance in s m^-1^. Because *T_0_* is difficult to obtain by thermal infrared remote sensing, we used surface temperature *T_s_* instead of *T_0_*. The sensible heat flux might be overestimated when the surface temperature is high. Here, *r_a_* is calculated using the following expression [9]:

(4) $$r_{a}=\frac{\left[\ln\left(\frac{z_{u}-d_{0}}{z_{0M}}\right)-\psi_{M}\right]\;\left[\ln\left(\frac{z_{t}-d_{0}}{z_{0H}}\right)-\psi_{H}\right]}{k^{2}u}$$

where *z_u_* and *z_t_* are the respective heights at which the wind speed *u* (in m s^-1^) and atmospheric temperature are measured, *d_0_* is the displacement height, and *z_0M_* and *z_0H_* are the roughness lengths for momentum and heat transport, respectively. All heights and roughness lengths are in meters. *Ψ_M_* and *Ψ_H_* are stability correction functions for momentum and heat, which depend on the Monin-Obukhov length [9], and *k* is von Karman's constant (= 0.4). *z_u_*, *z_t_* and *d_0_* are obtained by the methods described in Kato and Yamaguchi [1]. Roughness lengths show the height at which the neutral wind profile is extrapolated to a zero wind speed [10] and depend on obstacle heights and spacing on land surface. Some methods have been offered to estimate surface roughness from obstacle height, area and arrangement [11-13]. However, it is quite difficult to obtain requisite parameters to estimate roughness length through a wide area. Although we used ASTER DEM data, about 15 m of vertical accuracy and 15m of spatial resolution are not enough to evaluate obstacle height and arrangement. In this study, typical values of roughness lengths, *z_0M_* and *z_0H_*, are alternatively used for the selected surface types [9, 14 -21], as shown in Table 1. The surface types were obtained from a land cover map, as described in Section 4. We applied a logarithmic averaging procedure [22] in order to set the roughness length in each resultant pixel, which is 90m resolution according to ASTER TIR data in this particular case. Although spatial resolution of ASTER TIR data is 90m, the roughness length varies with location in this study.

Latent heat flux for evapotranspiration is expressed as

(5) $$LE=\frac{\rho C_{p}}{\gamma }\cdot \frac{e_{s}^{*}-e_{a}}{r_{a}+r_{s}}$$

where e_s_* is the saturation water vapor pressure in hPa at the surface temperature, *e_a_* is the atmospheric water vapor pressure in hPa, *γ*is the psychrometric constant in hPa K^-1^, and *r_s_* is the stomatal resistance in s m^-1^. Stomatal resistance is calculated based on the Jarvis-type scheme [23] simplified by Nishida et al. [24]:

(6) $$\frac{1}{r_{s}}=\frac{f_{1}\left(T_{a}\right)f_{2}\left(\text{PAR}\right)}{r_{\text{sMIN}}}+\frac{1}{r_{\text{cuticle}}}$$

where *PAR* is the photosynthetic active radiation in W m^-2^, *r_sMIN_* is the minimum stomatal resistance in s m^-1^, and *r_cuticle_* is the canopy resistance related to the diffusion through the cuticle layer of leaves in s m^-1^. *f*_1_ and *f*_2_ are estimated by the equations proposed by Jarvis [23] and Nishida *et al.* [24]. As well as roughness lengths, *r_sMIN_* was alternatively determined for each vegetation type that was decided upon and interpolated via surface classification based on the reference of Kelliher *et al.* [25] and Noilhan and Lacarrère [22]. The values of *r_sMIN_* used in this particular case are listed in Table 1. Latent heat flux is calculated in proportion to the fraction of water, vegetation type, and pervious surfaces with each *r_sMIN_* in one pixel.

## 3. Study Area and Data Used

**Figure 1.**
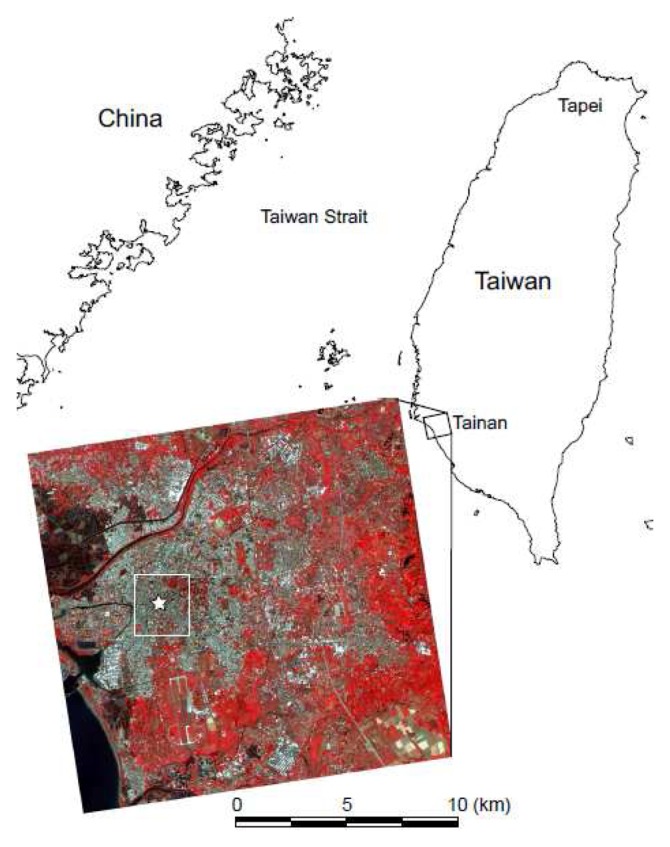
Location of the study area: Tainan City, Taiwan and Formosat-2 false color image. The star on Formosat-2 image represents the location of the meteorological station. The white rectangle is the area shown in Figures 3 and 6.

We selected an area of approximately 280 km^2^ covering Tainan City, Taiwan, for our study area, as Lin *et al.* [26] reported 3.4°C of heat island intensity at midnight in this area (Figure 1). Tainan currently has a population of about 750, 000 and is the fourth largest city of Taiwan. The present study area covers five of six districts in Tainan. The remaining northernmost district, An Nan, was excluded in this study because most of the area is used for agriculture. A large portion of the urban areas are located on the plain at the left side of the study area. The other areas are occupied by agricultural use. In the upper left of the study area, there are maintained fish farms which are widely distributed on the northwestern coast of Tainan City.

### 3.1. Satellite Data

The Advanced Spaceborne Thermal Emission and Reflection radiometer (ASTER) is an instrument onboard the Terra satellite. The spatial resolutions of the sensor are 15 m for the VNIR, 30 m for the shortwave infrared radiometer (SWIR), and 90 m for the TIR, respectively. Band numbers of each radiometer are 3 for VNIR, 6 for SWIR and 5 for TIR. The following ASTER data products were used: surface kinetic temperature, surface spectral emissivity, VNIR surface spectral reflectance, SWIR surface spectral reflectance, and the relative digital elevation model (DEM).

Formosat-2 is the Taiwanese satellite launched by the National Space Organization (NSPO), Taiwan, in 2004. Spatial resolution of Formosat-2 data is 8 m for the 4-band multispectral mode and 2 m for the panchromatic mode, respectively. Formosat-2 can revisit the same areas in one-day intervals. The authors used only multispectral data at the 8 m spatial resolution for the purpose of classifying the surface cover types.

We used ASTER data acquired in the daytime of March 6, 2001 and Formosat-2 data acquired on July 12, 2004, respectively, to estimate the heat balance in Tainan City.

### 3.2. Meteorological Data

The authors used the ground meteorological data acquired at the meteorological station in Tainan City, managed by the Southern Region Weather Center, Central Weather Bureau of Taiwan. The weather station is located in the center of the city, as shown in Figure 1. Meteorological observations come from a standard site but which is surrounded by dense urbanized surfaces and thus it is expected that the observations are representative of canopy layer conditions in the urban area. In the present study, the data used were solar radiation, wind speed, relative humidity, air-pressure and atmospheric temperature acquired at 1100 TST on March 6, 2001, according to the acquisition date of the ASTER data.

The meteorological data are summarized in Table 2. The interpolation method is basically the same as that of Kato and Yamaguchi [2]. Because there is only one meteorological observation site and the study area is relatively small, we assumed that atmospheric temperature and air-pressure, at 0m ASL, are the same throughout the study area. Extrapolations of these parameters for each pixel including altitudinal corrections were applied based on the environmental lapse rate with ASTER DEM data. Solar radiation, wind speed and relative humidity were assumed to be constant throughout the study area.

## 4. Surface Classification

In order to estimate surface heat fluxes, the authors needed to interpolate the roughness length for sensible and latent heat fluxes, and the minimum stomatal resistance for latent heat flux, respectively, based on surface types as mentioned in Section 2. Because of that, we wanted to classify the surfaces according to vegetation types, and density and height of buildings. In the present study, the authors classified surface types from satellite data by the combined methods of the maximum likelihood, decision tree and manual classification in order to separate surface coverage having similar spectral patterns. First, we classify the surface types in more than 30 categories by the maximum likelihood method. The categories are too numerous for the following analysis because they are based on not only surface coverage but also their spectral pattern. Therefore, they are combined into 8 categories: buildings, roads, water, bare soil, short grass (e.g., lawn), tall grass (e.g., paddy field), bushes, and forests. However, in the case of ASTER, bush could not be distinguished from the other vegetation types because of the limitations of band numbers and spatial resolution. In the case of Formosat-2, misclassification often occurred between water, sparse vegetation, dark soil and shadow because of the similarity of their spectral patterns. We extracted areas selected as water, and then classified them by the decision tree method. The decision tree was constructed by the following steps: 1) separate sparse vegetation by NDVI; 2) separate dark soil by NIR spectral pattern (band 4 of Formosat-2); and, 3) separate pavement and water by the sum of the DN values of band 1, 2, 3 and 4 of Formosat-2. We manually determined the thresholds for each step. Step 2 is based on the spectral characteristic of water that is low in the NIR region. Step 3 is based on the fact that reflectance of water is low for all spectral bands. After the decision tree procedure, there were still small areas suffering misclassifications. It is difficult to distinguish between water surfaces and shaded areas from their spectral patterns because the ranges of DN values of each band are extremely small. However, the areas of shadows are usually much smaller than those of water bodies, therefore we could manually distinguish them by comparison with the other maps.

We applied the above mentioned procedures to Formosat-2 multispectral data. On the other hand, for comparison purposes, we used the Maximum Likelihood method for ASTER VNIR data. Since the data acquisition dates of Formosat-2 and ASTER data are different, surface coverage is different in some agricultural areas. In the present study, our primary purpose of surface classification is to obtain a detailed surface coverage to estimate the heat fluxes on March 6, 2001. In order to modify the changed surface coverage between the two dates, we replaced the classification results by Formosat-2 data in some agricultural areas where surface types were different from those on the classification map produced from ASTER data.

## 5. Comparison of Surface Classification Maps

**Figure 2.**
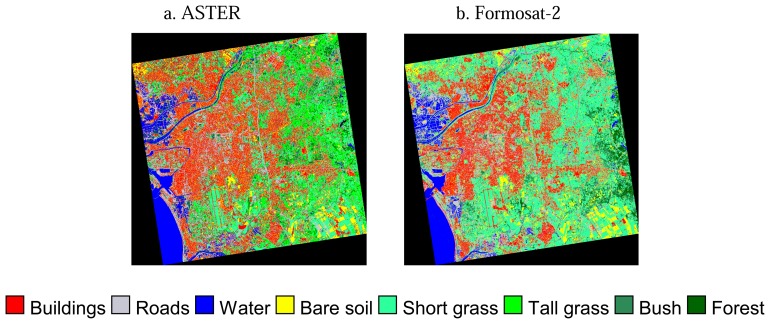
Surface classification maps derived from (a) ASTER and (b) Formosat-2 data.

Surface classification maps derived from ASTER and Formosat-2 data are shown in Figure 2, while the pixel numbers of each surface type are compared in Table 3. In the case of the classified results by ASTER, the buildings category shows the largest area. However, more areas were classified as short grass than urban areas on the Formosat-2 image. The areas classified as tall grass by ASTER changes to short grass in the case of Formosat-2 because of the similar spectral patterns of these two types. Because of the higher spatial resolution and additional blue band of Formosat-2, short grass in the parks in urban areas could be distinguished from building roofs. In fact, when surface types are classified without band 1 of Formosat-2, the areas of short grass were partly classified as buildings. The areas classified as road increased about 39% because of the higher spatial resolution of Formosat-2. Because usually a two-track road is wider than 8m and because of the spatial resolution of Formosat-2, narrow roads are detected relatively well. 49% and 20% of the areas of road were originally classified as road and buildings by ASTER, respectively. These results imply that the surface classification map by Formosat-2 distinguishes roads between buildings more clearly. In the case of ASTER, only some major roads are separated from buildings, but the areas of roads are overestimated because pixels are classified as road even if they contain roads with a width of less than 15m.

Both results show different spatial distributions of vegetation because it is difficult to distinguish vegetation types between grasses, while seasonal differences change the colors of vegetation. Similarly, because the spectral patterns of bare soil and building roofs are similar in this area, considerable areas of bare soil are misclassified as buildings and vice versa in both ASTER and Formosat-2 data.

In addition to misclassification between buildings and bare soil, there are some paved areas misclassified as buildings in both classification maps from ASTER and Formosat-2 because of their similar spectral patterns. For example, the considerable areas of the runways of Tainan airport, which is located in the lower left of the study area, are classified as bare soil and buildings rather than as roads by ASTER and Formosat-2, respectively. Since it is difficult to distinguish these surface types with similar spectral patterns by the supervised classification methods and to correct them manually through the whole area, the authors used these classification results as input data to the heat balance analysis without further correction.

## 6. Results of Heat Balance Analysis and Discussion

### 6.1. Comparison between the Heat Fluxes from ASTER and Formosat-2

Figures 4 and 5 show heat fluxes estimated based on surface classification maps from ASTER and Formosat-2, respectively. *R_n_* is shown only in Figure 4 because it is estimated without surface types. As mentioned in Section 2, the roughness length for *H* and *LE* and the stomatal resistance for *LE* are interpolated according to the surface classification map. Moreover, *ΔG* is also affected by the surface types because it is estimated by the residual of the other heat fluxes.

**Figure 3.**
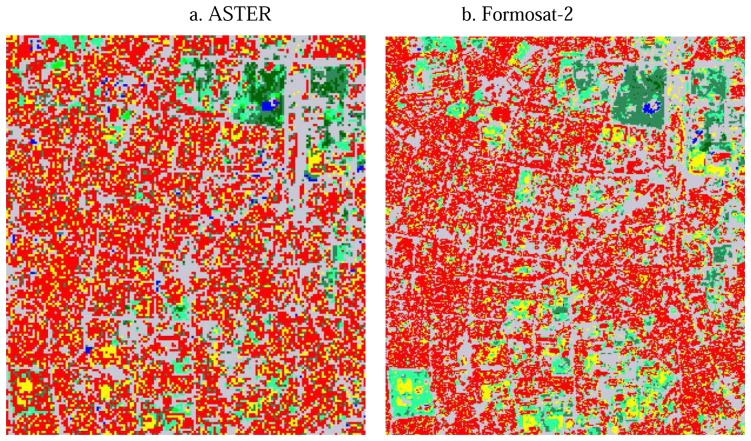
Surface classification maps in a portion of the urban area derived from (a) ASTER and (b) Formosat-2. Color legend is the same as Figure 2.

**Figure 4.**
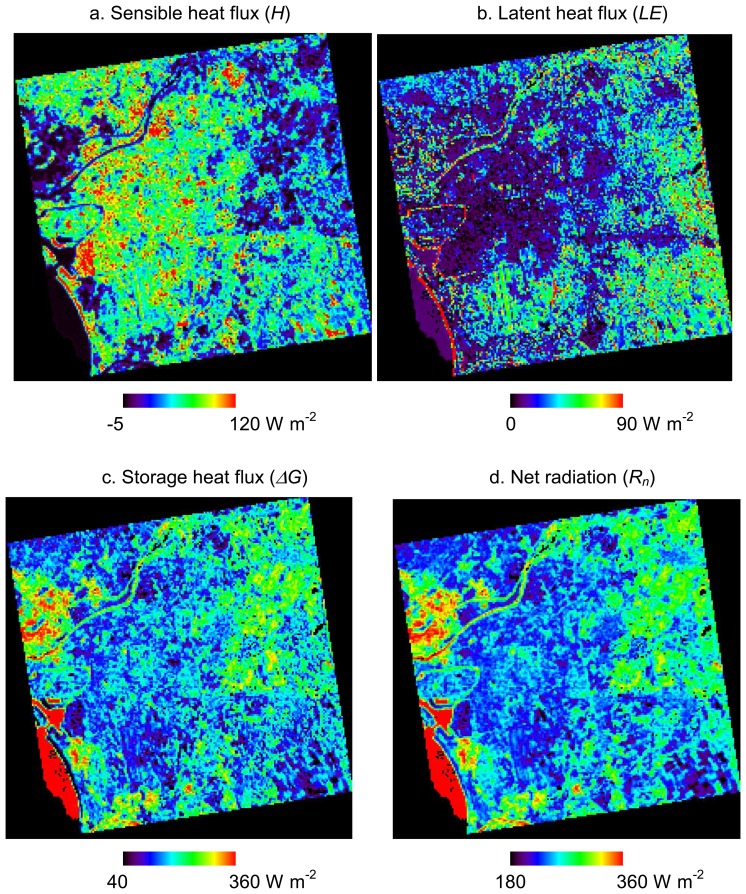
Distributions of (a) sensible heat flux *H*, (b) latent heat flux *LE*, (c) storage heat flux *ΔG*, and (d) net radiation *R_n_* estimated by using a surface classification map from ASTER data.

**Figure 5.**
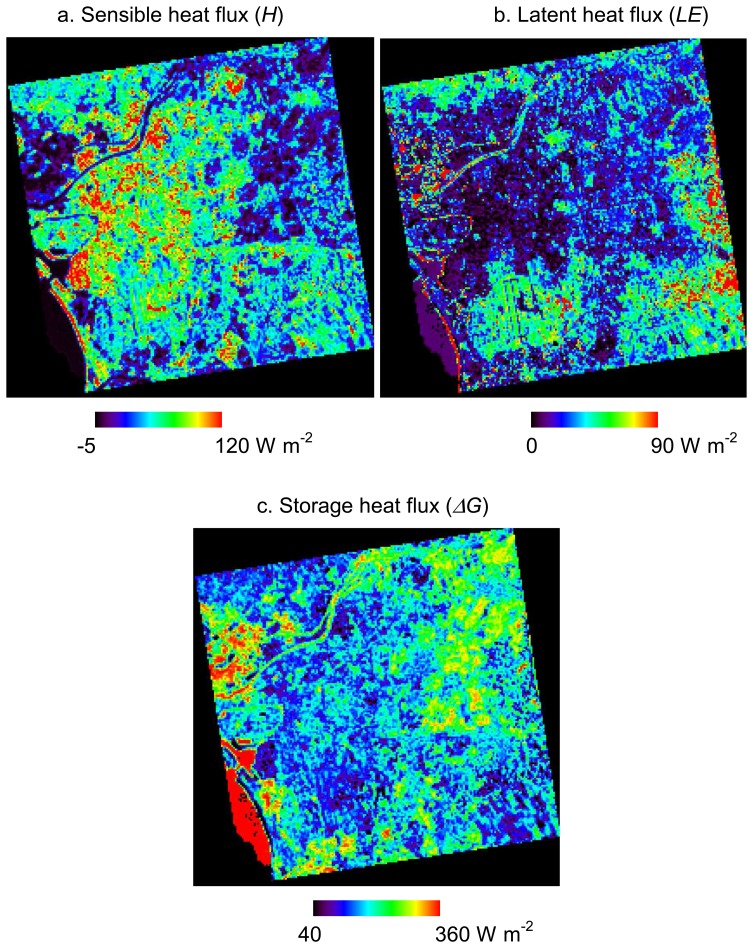
Distributions of (a) sensible heat flux *H*, (b) latent heat flux *LE*, and (c) storage heat flux *ΔG* estimated by using a surface classification map from Formosat-2 data.

**Figure 6.**
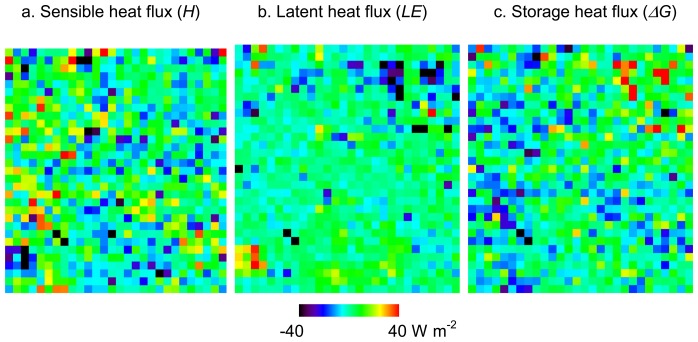
Heat flux differences between the results derived from ASTER and Formosat-2 in a part of the urban area: (a) sensible heat *H*, (b) latent heat *LE*, and (c) storage heat flux *ΔG*.

Table 4 summarizes the differences between the results of heat fluxes from ASTER and Formosat2 for the whole study area. We subtracted the heat fluxes from ASTER from those of Formosat-2 for each pixel. Based on the surface classification map from ASTER, we estimated the averages and standard deviations according to each surface type. Because the spatial resolutions of surface classification maps and resultant heat fluxes are different, we chose only the pixels of heat fluxes occupied by more than 60% of the same surface type from ASTER and where more than 50 % of the surface type was changed by Formosat-2. These requirements are determined to represent surface uniformity and to evaluate a large number of pixels with enhanced surface types, respectively. Although the purpose of this study is to assess the effect of mixed pixel in surface classification to heat flux estimation, we determined the first requirement in order to show tendencies for each surface type in Table 4. The differences of heat fluxes between the results from ASTER and Formosat-2 are significant in certain areas. For instance, Figure 6 shows the heat flux differences (Fluxes from Formosat-2 – Fluxes from ASTER) on a portion of the urban area containing improved classification results by Formosat-2, which is the same area as Figure 3. *H* decreased in the areas where the surface type was classified as buildings. Because the area of roads increases and that of buildings decreases, as mentioned in Section 5, roughness lengths on such areas decrease and, hence, *H* decreases. *H* also decreased in the areas originally classified as roads. This was caused by the surrounding building areas being changed to road by Formosat-2. On the other hand, *H* increased in some areas originally classified as bare soil. This is because many areas are misclassified by ASTER and reclassified as buildings by Formosat-2. In a few areas, *H* increased or decreased more than 100 Wm^-2^. We consider these results as errors attributed to apparent misclassification by ASTER or Formosat-2 because such large differences in heat fluxes are not realistic. The bare soil areas misclassified as buildings by ASTER show such extremely high *H*. On the other hand, the areas with large increases of *H* corresponded to the forest extracted by Formosat-2. Because roughness length should be determined not from surface types but obstacle heights and spacing, error can occur in forest areas misclassified as grasses. Although there are still possibility of misclassification of ASTER and Formosat-2 in the other areas, it is obvious that classification by Formosat-2 improve the estimation of *H* in bare soil and forest areas.

*LE* decreased in most of the areas classified as vegetation by ASTER except for short grass areas, and corresponded to the reclassification of surface types to another vegetation type which had less active transpiration. Because surface types in many areas have been changed from forest to bush and short grass by Formosat-2 data, *LE* especially decreased in forest areas. On the other hand, *LE* increased in short grass areas because short grass has the least active transpiration and reclassified as different vegetation types. *LE* slightly increased in the areas originally classified as roads and buildings by ASTER. Increases of *LE* should correspond to an increase of areas of vegetation and water bodies. In fact, the areas actually corresponded to the green vegetation in urban areas and fish farms. Similar to *H*, the large increase of *LE* in some small areas was affected by the misclassification caused by the differences of data acquisition dates and the seasons when the ASTER and Formosat-2 data were taken. As mentioned in Section 4, we tried to modify the surface type changes between two maps, but there is still error induced by misclassification. In order to solve this problem, it would be necessary to use data acquired on the same or closer dates or, at least, in the same season. Except for such erroneous places, most of the areas with increased *LE* in Formosat-2 data were caused by the increased detection of small vegetation and the change of vegetation types that have more active transpiration. In urban areas, *LE* increases in the areas with increased detection of small vegetation and decreases in the areas with reduced overestimation of vegetation and pervious surfaces.

The differences of *ΔG* are the total of the differences of *H* and *LE*. In most of the areas, decreases in both of *H* and *LE* result in an increase in *ΔG*. The largest decrease in *ΔG*, more than 100 W m^-2^, appeared in the areas reclassified as forest and water because of their higher *LE*. On the contrary, the largest *ΔG* increase corresponds to the area where surface types changed from forest and water to other surface types. In some of the vegetation areas, an increase in *LE* and a decrease in *H* are balanced out which results in a slight decrease in *ΔG*. On the other hand, a decrease in *ΔG* is a result of increased *H* and decreased *LE* in urban areas. It is caused by the reclassification of surface types from bare soil to buildings.

As a whole, heat fluxes are estimated in a reasonable range of values derived from both ASTER and Formosat-2 data. However, the land surface can be classified in higher spatial resolution by Formosat-2 data as mentioned in Section 5, and thus we can improve the heat flux estimation accordingly.

### 6.2. Sensitivity Analysis

**Figure 7.**
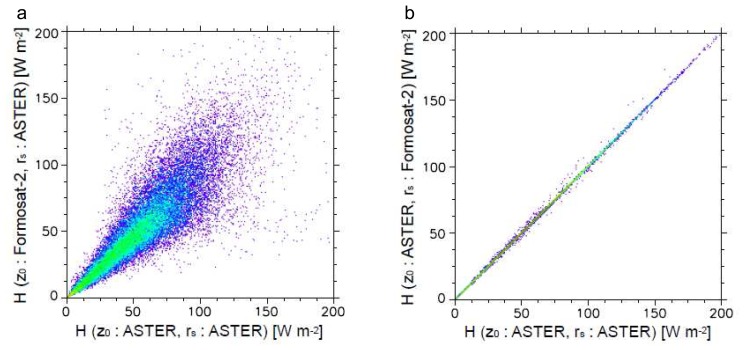
Scatterplots of sensible heat flux, *H*, estimated from ASTER vs. a) sensible heat flux estimated based on the roughness length from Formosat-2 and the minimum stomatal resistance from ASTER, and b) sensible heat flux estimated based on the roughness length from ASTER and the minimum stomatal resistance from Formosat-2.

**Figure 8.**
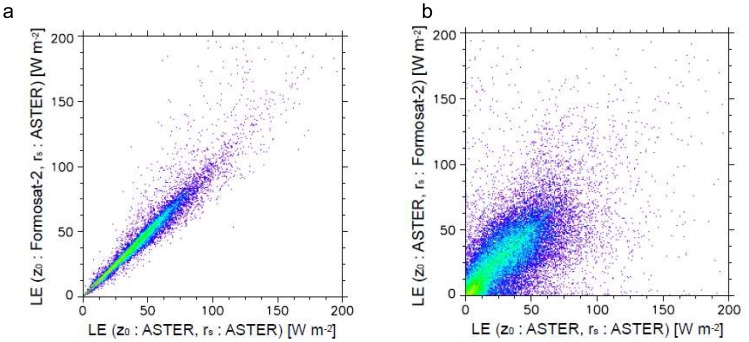
Scatterplots of latent heat flux, *LE*, estimated from ASTER vs. a) latent heat flux estimated based on the roughness length from Formosat-2 and the minimum stomatal resistance from ASTER, and b) latent heat flux estimated based on the roughness length from ASTER and the minimum stomatal resistance from Formosat-2.

**Figure 9.**
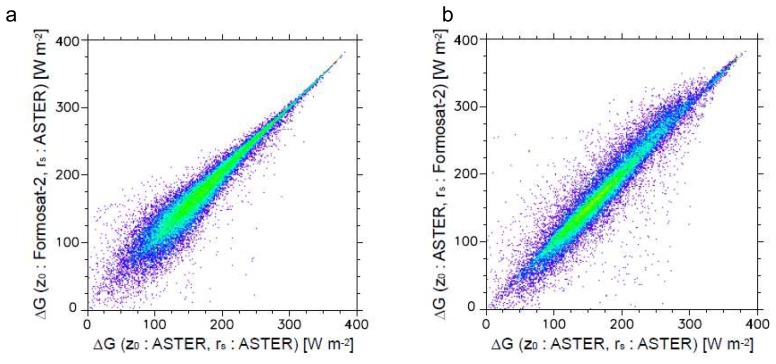
Scatterplots of storage heat flux, *ΔG*, estimated from ASTER vs. a) storage heat flux estimated based on the roughness length from Formosat-2 and the minimum stomatal resistance from ASTER, and b) storage heat flux estimated based on the roughness length from ASTER and the minimum stomatal resistance from Formosat-2.

In order to individually assess the effects by the roughness length and the stomatal resistance to heat flux estimation, we estimated heat fluxes according to the following two cases: 1) heat fluxes estimated based on the roughness length from Formosat-2 and the minimum stomatal resistance from ASTER, and 2) heat fluxes estimated based on the roughness length from ASTER and the minimum stomatal resistance from Formosat-2, respectively. The values of the roughness length and the stomatal resistance were determined according to the values shown in Table 1. Figure 7, 8 and 9 show the scatterplots between these results with the heat fluxes from ASTER data.

As a whole, moderate or high correlations were found for all cases. Because *H* is estimated without *r_s_* (Equation (3)), the correlation between *H* from ASTER and *H* based on *r_s_* from Formosat-2 is quite high (*r* = 0.9999). However, because *ψ_M_* and *ψ_H_* are dependent on the values of *H* and *LE* themselves [9], and are iteratively estimated in this study, slight deviations of *H* based on the replacement of *r_s_* values were found in Figure 7b. On the other hand, Figure 7a shows significant deviation caused by the altering of the value of *z_0_* (*r* = 0.8897), although many points are concentrated on the 1 : 1 line. The values of *H* are highly deviated in the area with higher *H* values. There are mainly three factors to increase *H* values in Equation (3); namely, wind speed, temperature difference between surface and atmosphere, and the roughness length. Since we applied a fixed value of wind speed in this analysis, this result indicates that the value of the roughness length is much more important for the places with higher temperature differences between surface and atmosphere. Because higher surface temperature in urban areas generally results in higher sensible heat flux than vegetation areas, the roughness length is one of the most important factors in estimating sensible heat flux in urban areas.

Contrary to *H*, the stomatal resistance affects *LE* more than the roughness length (Figure 8), despite *LE* being dependent on both parameters [Equation (5)]. The correlation is high between *LE* estimated by ASTER and *LE* from *z_0_* based on Formosat-2 (*r* = 0.9152). On the other hand, *LE* shows large deviations caused by the difference of the *r_s_* value (*r* = 0.5212). These results suggest that it is important to accurately determine *r_s_* in order to estimate *LE* when applying the present estimation method. On the other hand, *LE* is less dependent on the roughness length than the estimation of *H*. Distribution of latent heat from net radiation is dominated more by the potential of evapotranspiration than surface aerodynamic properties.

Although *ΔG* is estimated from *H* and *LE* as described in Equation (2), *ΔG* from ASTER correlates with both *ΔG* according to *z_0_* from Formosat-2 and *ΔG* according to *r_s_* from Formosat-2 (*r* values are 0.9426 and 0.9213, respectively). These results are caused by the fact that *ΔG* depends on *R_n_* more than *H* and *LE* in this particular case; namely the influence of the intensity of solar radiation to heat storage on land surface is larger than surface roughness and transpiration of vegetation. Hence, *ΔG* can be estimated reasonably well when the surface parameters are obtained with the spatial resolution of ASTER VNIR.

### 6.3. Spatial Pattern of Heat Fluxes

In this section, the following discussion is based on the results from Formosat-2 data because the purpose of this study was the combined use of ASTER and Formosat-2 data. In urban areas of Tainan, *H* and *ΔG* are about 80 to 170 Wm^-2^ and 130 to 200 Wm^-2^, and correspond to 40 to 70% and 60 to 80% of *R_n_*, respectively. These results are reasonable in comparison with previously published in situ measurement data. For example, based on the observation by Oke *et al.* [3], *H* and *ΔG* occupy 38% and 58% of *R_n_* in winter of an urban center in Mexico City, Mexico. In the case of the residential area studied by Moriwaki and Kanda [7], *H* and *ΔG* were 49% and 26% in summer and 36% and 61% in winter in Tokyo, Japan, respectively. These published data of ground measurements might not be suitable for direct comparison with our results because they are daytime mean values and measured in different cities. However, the authors take this comparison as meaningful because the surface heat balance primarily depends on surface materials and local aerodynamic conditions.

In urban areas, even if using higher resolution surface types by Formosat-2, *LE* from vegetation is quite small, namely about 10 W m^-2^, though, in general, latent heat flux varies significantly depending on the amount of vegetation. Because, in the present study, *LE* was estimated according to the intra-pixel area of vegetation, there might be some important meteorological and plant physiological phenomena which we did not consider. For example, the oasis effect by advection has been reported to increase evaporation from limited vegetation in urban areas [7]. Moreover, because *LE* was estimated to not represent water vapor transport in the canopy layer but evapotranspiration on the land surface, it is not always comparable with ground measurements.

Kato and Yamaguchi noted the diurnal change of heat balance in urban areas, namely higher *ΔG* in the daytime and higher negative *ΔG* at nighttime, caused by high thermal inertia in the urban center of Nagoya, Japan [2]. However, in the case of Tainan City, *ΔG* in dense building areas is smaller than *ΔG* in green vegetation areas. Differences of building materials are considered as one of the causes of these differences of spatial patterns of heat fluxes. In the case of Nagoya, Japan, the central part of the city is occupied by high-rise buildings constructed by bright concrete and wide glass windows. The landscape of the city center of Nagoya contrasts with surrounding residential areas of low houses with dark tile roofs. On the other hand, both the buildings for businesses and residences are constructed of concrete in Taiwan. Hence, there is a weak contrast of heat balances between urban and residential areas. Moreover, because many of the buildings have roofs made of steel with zinc plating in Tainan, the heat capacity of the roofs are smaller than those of concrete roofs. Therefore, *ΔG* values in buildings are small in the daytime. Another significance is high *ΔG* in green vegetation in urban areas, which corresponds to low *LE*, in contrast with low *ΔG* and high *LE* in arboreous mountain areas. Because there are sparse trees and spacious lawns in such areas, this result implies that the density of vegetation, namely transpiration activity, has a significant influence for temperature decreases. For further discussion, it is necessary to analyze the heat balance at nighttime for Tainan City.

## 7. Conclusions

Surface heat balance in Tainan City, Taiwan, on March 6, 2001 was estimated by ASTER and Formosat-2 data. Formosat-2 data were used to overcome the limitation of low spatial resolution of ASTER VNIR data. By using Formosat-2 data, we could reduce misclassifications attributed to mixed pixels and less spectral range. In particular, roads and small vegetation areas are easily distinguished from buildings, so that heat fluxes in urban areas could be estimated more accurately.

There are small differences between the heat fluxes estimated from the surface classification maps by ASTER and Formosat-2. However, Formosat-2's higher spatial resolution of surface types improves the heat flux estimation in certain areas. Most of the improved results are due to decreases in *H* and *LE* and increases in *ΔG* caused by decreased overestimation of roughness lengths and vegetation areas. There are also increases and decreases of heat fluxes raised from misclassifications by Formosat-2. Such misclassifications were caused by land use changes which occurred in an interval of about three years between the acquisition dates of ASTER and Formosat-2 data. In order to mitigate misclassification, it is desirable to use satellite data taken on the same or closer dates or, at least, in the same season. In addition, the authors concluded that surface classification maps are appropriate qualitatively by the visual comparison and the tendency of results of heat flux analysis. However, it is necessary to verify the accuracy of surface classification with independent data and testing in order to estimate quantitative accuracy.

Heat fluxes estimated in this study are comparable with previous in situ measurement data in other cities. However, *ΔG* in urban areas has no contrast with *ΔG* in surrounding residential areas. This result disagrees with the general trend in urban areas with a high heat capacity. We assume this is because of the homogeneity of building materials in both business and residential areas, and the many roofs that are made of steel with zinc plating, which has a small heat capacity in Tainan. It would be interesting to compare heat balances in other cities belonging to different climates and to consider the influence of construction materials.

The present study showed that the 8m spatial resolution and 4 spectral bands of Formosat-2 can improve the classification of surface types for input data to estimate the heat balance in urban areas as compared with the 15m spatial resolution and 3 spectral bands of ASTER VNIR data. As a result of the level of sensitivity in this study, the vegetation types classified in higher spatial resolution and accuracy by Formosat-2 increased the accuracy of latent heat flux estimation. This is one of the future issues to examine -which spatial resolution is the most suitable to, or high enough, for identifying vegetation areas and types for heat balance analysis. Interestingly, there are still higher spatial resolution data from satellite remote sensing; for example, even Formosat-2 has a 2m panchromatic band. On the other hand, all of the sensible, latent and storage heat fluxes are slightly improved by the roughness length in higher spatial resolution. Interpolating the roughness lengths from surface types has a limitation in obtaining accurate values because roughness length is the index of obstacle height and spacing, not land coverage. If the surface elevation can be directly measured via satellite in high resolution, it will be possible to estimate much more accurate roughness lengths and, consequently, heat fluxes.

## Figures and Tables

**Table 1.** Parameters fixed for surface coverage types.

**Surface type**	***z****_0M_* **(m)**	***kβ*^-1^ = ln(*z_0M_***/***z_0H_*)**	***r_sMIN_* (s m^-1^)**
Building	0.5	7.0	-
Road	0.05	5.1	-
Water	0.00003	-1	0
Bare soil	0.001	5.1	2500
Short grass	0.01	5.1	1000
Tall grass	0.1	5.1	625
Bush	0.1	5.1	1000
Forest	0.5	7.0	180

**Table 2.** Summary of the meteorological conditions of the analysis in Tainan City at 1100 TST on March 6, 2001.

**Variable (Units)**	**Value**
Shortwave radiation (W m^-2^)	453
Wind speed (m s^-1^)	0.9
Atmospheric temperature (K)	300.6
Air pressure (hPa)	1016.4
Relative humidity (%)	50

**Table 3.** Pixel numbers and percentages for each surface type category derived from ASTER and Formosat-2 data.

**Surface type**	**ASTER**		**Formosat-2**	

	Pixel number	Percentage (%)	Pixel number	Percentage (%)
Buildings	1146475	26.7	801997	18.6
Roads	506137	11.7	702220	16.3
Water	315051	7.3	292800	6.8
Bare soil	469174	10.9	514532	11.9
Short grass	1245561	28.9	1586204	36.8
Tall grass	482919	11.2	12630	0.3
Bush	0	0	315793	7.3
Forest	149294	3.5	88435	2.0

Total pixel numbers are standardized to that of Formosat-2.

**Table 4.** Averages and standard deviations of heat flux differences derived from ASTER and Formosat-2 data (Fluxes from Formosat-2 – Fluxes from ASTER).

**Surface type**	***H*(W m^-2^)**		***LE*(W m^-2^)**		*Δ****G*(W m^-2^)**	

	Average	Standard deviation	Average	Standard deviation	Average	Standard deviation
Building	-47	29	33	33	14	37
Road	-5	23	32	54	-27	44
Water	10	24	-59	87	49	65
Bare soil	47	30	-13	12	-34	24
Short grass	18	14	12	34	-31	36
Tall grass	-5	10	-12	24	17	31
Forest	-6	9	-58	24	64	31

Surface types are based on the map derived from ASTER.
